# Haplotype analysis of endothelial nitric oxide synthase (NOS3) genetic variants and metabolic syndrome in healthy subjects and schizophrenia patients

**DOI:** 10.1038/s41366-018-0124-z

**Published:** 2018-06-15

**Authors:** Nikolai Fattakhov, Liudmila Smirnova, Dmitriy Atochin, Daria Parshukova, Daria Skuratovskaia, Quinn Painter, Pavel Zatolokin, Arkadiy Semke, Larisa Litvinova, Svetlana Ivanova

**Affiliations:** 1 Institute of Medicine, Immanuel Kant Baltic Federal University, Kaliningrad, Russia; 2 Mental Health Research Institute, Tomsk National Research Medical Center of the Russian Academy of Sciences, Tomsk, Russia; 3 Cardiovascular Research Center, Massachusetts General Hospital, Boston, MA USA; 4 RASA Center in Tomsk, National Research Tomsk Polytechnic University, Tomsk, Russia; 5 Department of Psychiatry, Psychosomatic Medicine and Psychotherapy, University Hospital of Frankfurt, Frankfurt am Main, Germany; 6 Department of Reconstructive and Endoscopic Surgery, Kaliningrad Regional Hospital, Kaliningrad, Russia; 7 Department of Ecology and Life Safety, National Research Tomsk Polytechnic University, Tomsk, Russia

## Abstract

**Background/objectives:**

The frequency of metabolic syndrome (MetS) is significantly higher in schizophrenia (SCH) patients, when compared to the general populatiotin. The goal of this study was to evaluate whether genetic variants T-786C (rs2070744), G894T (rs1799983) and C774T (rs1549758) in the endothelial nitric oxide (NOS3) gene and/or their haplotypes could be associated with the risk of MetS in SCH patients or healthy subjects from Russian population.

**Subjects/methods:**

We performed two case−control comparisons. NOS3 polymorphisms were genotyped in 70 SCH patients with MetS, 190 normal weight SCH patients, 155 MetS patients, and 100 healthy controls. MetS was defined as per the criteria proposed by the International Diabetes Federation (IDF). Anthropometric, clinical, biochemical parameters, and serum nitrite concentrations were measured in all samples. Haplotype frequency estimations and linkage disequilibrium measures were made using Haploview 4.2.

**Results:**

The higher C allele (*P* = 0.009) and lower TT genotype (*P* = 0.008) frequencies of T-786C polymorphism were found in SCH patients with MetS compared to those in normal weight SCH patients. SCH patients with MetS who were carriers of the T-786C TT genotype had lower serum total cholesterol levels in comparison to the CC genotype (*P* = 0.016). Furthermore, the 774T/894T haplotype was more frequent in non-SCH individuals with MetS compared to healthy controls (*P* = 0.0004, odds ratio = 2.18, 95% confidence interval 1.4–3.37). Conversely, the most common haplotype 774C/894G was less frequent in MetS patients than in healthy controls (*P* = 0.013, odds ratio = 0.61, 95% confidence interval 0.41–0.9).

**Conclusions:**

These results indicate that the NOS3 T-786C promoter polymorphism was closely associated with MetS risk in SCH patients. In addition, the haplotypes composed of G894T and C774T polymorphisms are associated with the MetS susceptibility in Russian population.

## Introduction

Metabolic syndrome (MetS) is described as a collection of interrelated factors that lead to an increase in risk for cardiovascular diseases and type 2 diabetes mellitus (T2DM). MetS has developed into a global health problem because of its rapidly increasing worldwide prevalence that ranges between 10 and 84% in various populations [1]. There are multiple definitions of MetS, and more recent criteria established by the IDF for practical worldwide use include abdominal obesity, as well as two of the following four factors: elevated fasting plasma glucose concentration or previously diagnosed T2DM, elevated triglyceride levels, high blood pressure and low levels of high-density lipoprotein (HDL) cholesterol [2]. Compared with the general population, individuals with SCH have significantly higher frequency rates of MetS, which remains the principal cause of cardiovascular mortality among this population [3, 4]. Several specific factors that may influence susceptibility to MetS in patients with SCH include antipsychotic medication and other severe impacts involving the mental illness itself [5–7]. Recent reviews showed that MetS in the general population, and MetS in SCH patients may have both shared and specific underlying genetic determinants [8, 9]. The identification of susceptibility genes and their functional variants corresponding with MetS risk might lead to effective interventions for its prevention and targeted treatment in both psychiatric and non-psychiatric populations.

The nitric oxide (NO) generated by the enzyme endothelial nitric oxide synthase (eNOS) regulates essential cardiovascular and metabolic functions [10]. The production of NO is impaired in patients with MetS features. This impairment can be related to reduced eNOS enzymatic activity and expression, alterations in eNOS phosphorylation, and eNOS uncoupling [11]. Mice that lacked the *NOS3* gene that codes for eNOS had certain cardiovascular risk factors which appear to imitate human MetS including hypertension, metabolic insulin resistance, and hyperlipidemia [12, 13]. In addition, partial as well as total deletion of the *NOS3* gene result in a significant deficiency in coronary vasodilation ability, providing yet more evidence that this gene may be accountable for the relationship between MetS and cardiovascular morbidity [14].

The *NOS3* gene is positioned within chromosomal region 7q36, which has shown a suggestive linkage to MetS-related traits in numerous genome-wide scans [15, 16]. Genetic variations in the nucleotide sequence at the promoter, the exons, and the intronic regions of the *NOS3* gene have been revealed. Amid the frequently identified NOS3 single nucleotide polymorphisms (SNPs), G894T (rs1799983) within exon 7 and T-786C (rs2070744) in the 5′-flanking region, are of particular interest because these SNPs may lead to modifications in gene expression, and may even influence interindividual differences related to the activity of the encoded protein [17, 18]. Variations such as these have been implicated in a number of studies investigating MetS, T2DM, and insulin resistance, but studies show inconsistent results [19–22]. These inconsistencies could perhaps be due to differences in sample size, age, MetS criteria, study design, and interethnic differences within the distribution of NOS3 genetic variants. A recent study showed that synonymous NOS3 SNP C774T in exon 6 (rs1549758) is associated with the development of microvascular and macrovascular complications of T2DM [23, 24]. However, the contribution of C774T nucleotide substitution in cardiometabolic factors still remains controversial [25]. The molecular mechanisms for how these three NOS3 SNPs might affect clinical outcomes are not fully investigated.

To date, no studies have inspected the association between NOS3 haplotypes and MetS in the Russian population. Furthermore, as far as we know, no study as of yet has examined the contribution of NOS3 SNPs to MetS risk in SCH patients. However, one study has been conducted recently which assesses the relationship between NOS3 G894T and T-786C SNPs and endothelial function in a group of SCH subjects who take antipsychotics [26]. The connection of NOS3 T-786C SNP with worse endothelial function was found only in SCH patients who did not have MetS. Hence, the intention of our study was to investigate potential associations of the NOS3 T-786C, G894T and C774T SNPs or their haplotypes with MetS risk in the Russian population and SCH patients. Additionally, associations of these SNPs with serum nitrite concentrations in SCH and non-SCH subjects with MetS were examined.

## Materials and methods

### Subjects

The first case−control comparison included 70 SCH patients with MetS defined according to the IDF definition (19 males, 51 females) and 190 normal weight SCH patients (122 males, 68 females). Normal weight was established as a body mass index (BMI) of 18.5−25 kg/m^2^. All SCH patients were recruited from three psychiatric hospitals in Kemerovo, Chita, and Tomsk areas in Siberia (Russia). Ethics approval to conduct this study was obtained from the Local Bioethics Committee of the Mental Health Research Institute (Tomsk, Russia). The main criteria for including the patients in both groups were a clinically verified diagnosis of SCH (ICD-10: F20), Russian ethnicity, Caucasian race identification, and the absence of organic or neurological disorders. Similar proportions of patients in both groups received treatment with atypical antipsychotic drugs (71% of SCH patients with MetS versus 74% normal weight SCH patients). Among the SCH patients with MetS, 16% received first-generation antipsychotics and 13% received combined treatment, whereas these percentages within the sample of normal weight SCH patients were 20 and 6%, respectively. The most frequently prescribed second-generation antipsychotics were risperidone, amisulpride, paliperidone, clozapine, olanzapine, and sertindole. The most common conventional antipsychotic was haloperidol.

For the second case−control comparison, we enrolled 155 Russian MetS patients (60 males, 95 females) from the Regional Clinical Hospital of the Kaliningrad Region and 100 healthy controls (48 males, 52 females) recruited from the same geographic region. Occurrence of MetS was established in accordance with the IDF criteria. All controls from the general population had normal weight and were free of infectious, chronic, and endocrine diseases. The Local Ethics Committee of Immanuel Kant Baltic Federal University (Kaliningrad, Russia) approved this study.

Both studies were conducted following the Declaration of Helsinki. Informed consent was acquired from each subject. To promote homogeneity in samples, all included SCH and non-SCH subjects belonged to Russian Caucasian population. Ethnicity was determined according to both the self-identification of each subject and the subject’s understanding of the ethnicity of their parents and four grandparents. For all cohorts, each participant was interviewed using a questionnaire to determine their cigarette smoking status (non-smoker or current smoker).

### Anthropometrical and biochemical assessments

Anthropometric measurements included waist circumference, hip circumference, waist-to-hip ratio, and BMI. The waist circumference was established as the smallest width between coastal margins and iliac crests, when taken at a minimum respiration. Hip circumference was obtained at the maximum extension of the buttocks while the participant was in standing position. Waist-to-hip ratio was found by dividing the waist by the hip values. BMI is the weight of the subject (kg) divided by height of the subject (m^2^).

For all participants, an overnight fasting venous blood sample was acquired during the same day as flow measurements. Biochemical studies of fasting blood glucose and serum lipid parameters (triglycerides, total cholesterol low-density lipoprotein (LDL) cholesterol and HDL-cholesterol) were carried out on a biochemical autoanalyzer CA-180 (Furuno Electric Co., Ltd., Hyogo, Japan) using DiaSys reagent kits (DiaSys Diagnostic Systems, Holzheim, Germany). The levels of nitrite were determined spectrophotometrically, based on the Griess reaction as described by Moshage et al. [27]. Briefly, 100 μl of serum samples were diluted fourfold with deionized water and deproteinized by adding 20 μl of zinc sulfate (1.85 M). After centrifugation (10,000 × *g*, 5 min), supernatants were transferred to wells of microtiter plates in duplicate, followed by the addition of 100 μl of Griess reagent. The absorbance was read at 540 nm after 10 min. The nitrite concentration in each sample was quantified by extrapolation from the sodium nitrite standard curve.

### Genotyping

All subjects were genotyped for the T-786C, G894T, and C774T SNPs in the *NOS3* gene using allele-specific real-time PCR. The cycling conditions for T-786C and C774T SNPs were 95 °C for 3 min, 50 cycles of 15 s at 95 °C, 40 s at 65 °C. The PCR protocol for G894T SNP included heating of the reaction mixture for 3 min at 95 °C and 50 amplification cycles performed as follows: 95 °C for 15 s, 63 °C for 40 s. Two PCR amplification reactions were set up for each sample. All PCRs were run on the LightCycler 480 Real-Time PCR System (Roche Diagnostics, Vienna, Austria). All three SNPs were genotyped with the SNP genotyping assays (Syntol JSC, Moscow, Russia). Primers and probes were designed and manufactured by Syntol JSC (Moscow, Russia).

### Statistical analysis

Statistical data analysis was executed using the Statistica version 10.0 (StatSoft, Tulsa, OK, USA). The Levene’s test was utilized to confirm the assumption of equal variances. The Kolmogorov–Smirnov test was applied to discern whether data followed normal distribution, and the significance of intergroup differences in continuous variables was determined using the independent *t* test. The gender variables were evaluated using Chi-square testing.

Departures of genotype frequencies from the Hardy−Weinberg proportions were evaluated using Chi-square testing. The allele, genotype, and haplotype frequencies of SNPs were compared between groups by using either Chi-square or Fisher’s exact test when deemed appropriate. One-way ANOVA followed by Fisher’s LSD post hoc test was performed to differentiate serum biochemical profiles depending on genotypes among SCH and non-SCH MetS patients.

Haplotype frequency estimations and LD measures were made with the program Haploview version 4.2 (Broad Institute, Cambridge, MA, USA). The solid spine of the LD method was used to estimate the haplotype block as a pairwise *D*′ value of greater than 0.7 between SNPs. Computation of the odds ratios and its 95% confidence intervals (CI) were performed using the statistical calculator on VassarStats website (http://vassarstats.net/). *A P* value <0.05 was deemed to be statistically significant.

## Results

The comparisons of clinical and biochemical variables among case samples and control samples are represented in Table 1. SCH patients with MetS had a higher BMI, waist circumference, waist-to-hip ratio, blood pressure when compared to normal weight SCH subjects. Compared to healthy controls, MetS patients had higher scores on all four anthropometric parameters, blood pressure, fasting glucose, triglycerides, and lower HDL-cholesterol levels. Serum nitrite content in SCH patients with MetS were threefold higher than those of normal weight SCH patients, while serum nitrite content in MetS patients was comparable to healthy controls. There was no difference in the smoking frequency among SCH patients with MetS compared with normal weight SCH subjects. Additionally, no significant variance appeared in the proportions of current smokers between MetS patients and healthy controls. When antidiabetic, antihypertensive, and cholesterol-lowering medication was compared between SCH patients with MetS and MetS patients, significant differences were found only for the proportion of patients taking oral antidiabetic agents (Table 2).

**Table 1:** Demographic characteristics of study participants

Variables	SCH patients with MetS (*n* = 70)	Normal weight SCH patients (*n* = 190)	*P* level	MetS patients (*n* = 155)	Healthy controls (*n* = 100)	*P* level
Age (years)	46.54 ± 13.08	43.8 ± 9.12	0.097	43.24 ± 8.44	41.23 ± 7.71	0.057
Female/male ratio	51 (72.86%)/19 (27.14%)	68 (35.79%)/122 (64.21%)	<0.001*	95 (61.29%)/60 (38.71%)	52 (52%)/48 (48%)	0.1427
BMI (kg/m^2^)	34.06 ± 4.13	21.56 ± 2.09	<0.001	40.87 ± 7.55	22.42 ± 2.15	<0.001
Waist circumference (cm)	103.31 ± 13.19	77.90 ± 6.71	<0.001	113.34 ± 15.52	76.38 ± 9.18	<0.001
Hip circumference (cm)	104 ± 20.58	95.17 ± 9.95	0.288	123.17 ± 14.88	95.5 ± 6.97	<0.001
Waist-to-hip ratio	0.98 ± 0.15	0.82 ± 0.04	0.008	0.93 ± 0.12	0.8 ± 0.07	<0.001
Systolic blood pressure (mm Hg)	136.10 ± 20.84	118.02 ± 16.03	<0.001	143.94 ± 24.55	112.67 ± 9.62	<0.001
Diastolic blood pressure (mm Hg)	86.76 ± 12.87	74.44 ± 13	<0.001	87.64 ± 14.76	71.5 ± 4.74	<0.001
Current cigarette smokers (*n*, %)	41 (58.57%)	94 (49.47%)	0.193	43 (27.74%)	23 (23%)	0.399
Fasting glucose (mmol/l)	5.55 ± 0.7	5.14 ± 0.65	0.093	6.66 ± 2.12	5.12 ± 0.35	<0.001
Total cholesterol (mmol/l)	5.67 ± 1.02	5.09 ± 1	0.115	4.41 ± 1.04	4.71 ± 0.43	0.139
HDL-cholesterol (mmol/l)	1.01 ± 0.27	1.2 ± 0.43	0.183	1.17 ± 0.29	1.49 ± 0.38	<0.001
LDL-cholesterol (mmol/l)	3.83 ± 0.82	3.34 ± 0.9	0.133	2.66 ± 0.86	2.7 ± 0.38	0.784
Triglycerides (mmol/l)	1.5 ± 0.5	1.15 ± 0.53	0.066	1.41 ± 0.77	0.86 ± 0.35	<0.001
Nitrites (µmol/l)	31.37 ± 18.31	11.82 ± 7.23	0.004	4.36 ± 1.8	4.09 ± 1.32	0.551

Data are expressed as mean  ±  s.d.

*SCH* schizophrenia, *MetS* metabolic syndrome, *BMI* body mass index, *HDL* high-density lipoprotein, *LDL* low-density lipoprotein

*P value calculated using Pearson’s Chi-square test

**Table 2:** Comparison of treatments for MetS in SCH and non-SCH subjects

Treatment	SCH patients with MetS (*n* = 70)	MetS patients (*n* = 155)	*P* level
Oral antidiabetic drugs	5 (7.14%)	30 (19.35%)	0.019
Antihypertensive drugs	29 (41.43%)	78 (50.32%)	0.216
Statins	28 (40%)	56 (36.13%)	0.578

Table 3 displays allele and genotype frequencies of NOS3 variants in all cases and controls. In all samples, genotype frequencies did not diverge from the Hardy−Weinberg proportions (all *P* > 0.05). We found that the T allele (*P* = 0.009) and the TT genotype (*P* = 0.008) of T-786C SNP were significantly less common among SCH patients with MetS than in normal weight SCH subjects. The C774T and G894T SNPs were not associated with MetS in SCH patients. However, the prevalence of T allele of the G894T (*P* = 0.002) and T allele of C774T (*P* = 0.003) was higher in MetS patients in comparison to healthy controls. The GT (*P* = 0.011) and TT genotypes (*P* = 0.0004) of G894T, as well as the heterozygous genotype of C774T (*P* = 0.035) were more common in MetS patients in comparison to healthy controls. Furthermore, frequencies of the GG genotype (*P* = 0.002) and CC genotype (*P* = 0.002), in turn, were less in MetS patients in comparison to healthy controls.

**Table 3:** Distribution of allele and genotype frequencies for the three NOS3 polymorphisms

Polymorphism	Genotype/Allele	SCH patients with MetS vs. SCH controls				Patients with MetS vs. healthy controls			
		SCH patients with MetS (*n* = 70)	Normal weight SCH patients (*n* = 190)	OR (95% CI)	*P* value	MetS patients (*n* = 155)	Healthy controls (*n* = 100)	OR (95% CI)	*P* value
T-786C (rs2070744)	TT	19 (27.1%)	86 (45.3%)	0.45 (0.25–0.82)	0.008	62 (40%)	40 (40%)	1 (0.6–1.67)	1
	TC	39 (55.7%)	84 (44.2%)	1.59 (0.91–2.76)	0.099	63 (40.6%)	48 (48%)	0.74 (0.45–1.23)	0.248
	CC	12 (17.1%)	20 (10.5%)	1.76 (0.81–3.82)	0.15	30 (19.4%)	12 (12%)	1.76 (0.85–3.63)	0.122
	T	77 (55%)	256 (67.4%)	0.59 (0.4–0.88)	0.009	187 (60.3%)	128 (64%)	0.86 (0.59–1.24)	0.404
	C	63 (45%)	124 (32.6%)	1.69 (1.14–2.51)		123 (39.7%)	72 (36%)	1.17 (0.81–1.69)	
G894T (rs1799983)	GG	39 (55.7%)	117 (61.6%)	0.78 (0.45–1.37)	0.392	61 (39.4%)	59 (59%)	0.45 (0.27–0.75)	0.002
	GT	27 (38.6%)	60 (31.6%)	1.36 (0.77–2.41)	0.289	75 (48.4%)	35 (35%)	1.74 (1.04–2.92)	0.011
	TT	4 (5.7%)	13 (6.8%)	0.83 (0.26–2.62)	0.744	19 (12.3%)	6 (6%)	2.19 (0.84–5.69)	0.0004
	G	105 (75%)	294 (77.4%)	0.88 (0.56–1.38)	0.571	197 (63.5%)	153 (76.5%)	0.54 (0.36–0.8)	0.002
	T	35 (25%)	86 (22.6%)	1.14 (0.73–1.79)		113 (36.5%)	47 (23.5%)	1.87 (1.25–2.79)	
C774T (rs1549758)	CC	38 (54.3%)	115 (60.5%)	0.77 (0.45–1.35)	0.364	68 (43.9%)	64 (64%)	0.44 (0.26–0.74)	0.002
	CT	26 (37.1%)	60 (31.6%)	1.28 (0.72–2.27)	0.398	73 (47.1%)	31 (31%)	1.98 (1.17–3.36)	0.035
	TT	6 (8.6%)	15 (7.9%)	1.09 (0.41–2.94)	0.859	14 (9%)	5 (5%)	1.89 (0.66–5.41)	0.101
	C	102 (72.9%)	290 (76.3%)	0.83 (0.54–1.3)	0.417	209 (67.4%)	159 (79.5%)	0.53 (0.35–0.81)	0.003
	T	38 (27.1%)	90 (23.7%)	1.2 (0.77–1.87)		101 (32.6%)	41 (20.5%)	1.87 (1.23–2.85)	

*SCH* schizophrenia, *MetS* metabolic syndrome, *OR* odds ratio, *CI* confidence interval

To explore how polymorphisms in the *NOS3* gene may be of pathophysiological importance when considering the MetS risk in SCH and non-SCH subjects, we evaluated NOS3 polymorphisms for the associations with MetS biochemical traits in these groups (Table 4). SCH patients with MetS with the TT genotype of the T-786C SNP showed lower levels of total cholesterol compared to carriers of CC genotype (*P* = 0.016). No significant effect of genotype in relation to serum nitrite level in these groups was discovered.

**Table 4:** Effects of NOS3 variants on serum biochemical levels in SCH and non-SCH patients with MetS

Variables	Polymorphism T-786C				Polymorphism G894T				Polymorphism C774T			
	TT	TC	CC	*P* value	GG	GT	TT	*P* value	CC	CT	TT	*P* value
**SCH patients with MetS (*n*** **= 70**)												
Fasting glucose (mmol/l)	5.61 ± 0.77	5.13 ± 0.85	5.15 ± 0.65	0.399	5.78 ± 1.05	5.14 ± 0.44	4.96 ± 0.51	0.12	5.7 ± 0.93	4.95 ± 0.41	5.10 ± 0.57	0.123
Total cholesterol (mmol/l)	4.45 ± 0.97*	5.33 ± 1.25	6.25 ± 0.35	0.046	5.69 ± 1.46	4.99 ± 1.08	5.09 ± 1.32	0.597	5.39 ± 1.23	5.03 ± 1.3	5.09 ± 1.32	0.854
HDL-cholesterol (mmol/l)	1.11 ± 0.34	0.87 ± 0.66	0.92 ± 0.06	0.602	0.86 ± 0.32	0.94 ± 0.42	1.17 ± 0.52	0.536	1 ± 0.41	0.78 ± 0.28	1.17 ± 0.52	0.367
LDL-cholesterol (mmol/l)	3.76 ± 0.97	3.02 ± 0.75	3.6 ± 1.18	0.318	4.15 ± 1.15	3.18 ± 0.61	3.24 ± 1.1	0.166	3.83 ± 0.97	3.12 ± 0.75	3.24 ± 1.1	0.338
Triglycerides (mmol/l)	1.33 ± 0.48	1.55 ± 0.65	1.3 ± 0.44	0.691	1.47 ± 0.68	1.44 ± 0.43	1.24 ± 0.56	0.75	1.4 ± 0.57	1.37 ± 0.57	1.43 ± 0.43	0.986
Nitrites (µmol/l)	32.73 ± 24.18	19.8 ± 17.79	40.16 ± 18.42	0.214	38.43 ± 22	23.73 ± 17.65	29.97 ± 26.23	0.476	39 ± 20.83	28.32 ± 20.42	22.33 ± 22.16	0.439
**MetS patients (*n*** = **155)**												
Fasting glucose (mmol/l)	5.96 ± 0.86	6.27 ± 0.77	6.37 ± 1.11	0.44	6.24 ± 0.92	6.11 ± 0.85	6.16 ± 0.94	0.954	6.27 ± 0.99	6.13 ± 0.7	5.96 ± 1.17	0.766
Total cholesterol (mmol/l)	4.15 ± 1.23	4.4 ± 0.98	4.86 ± 0.33	0.286	4.64 ± 1.11	4.25 ± 0.98	4.21 ± 1.37	0.491	4.71 ± 1.16	4.03 ± 0.82	4.65 ± 1.56	0.12
HDL-cholesterol (mmol/l)	1.19 ± 0.33	1.08 ± 0.23	1.21 ± 0.25	0.386	1.14 ± 0.2	1.19 ± 0.35	1.07 ± 0.21	0.578	1.22 ± 0.27	1.08 ± 0.29	1.15 ± 0.22	0.292
LDL-cholesterol (mmol/l)	2.57 ± 1.16	2.69 ± 0.71	2.89 ± 0.24	0.702	2.93 ± 1	2.47 ± 0.76	2,64 ± 0.98	0.295	2.94 ± 1.05	2.37 ± 0.59	2.95 ± 1.1	0.094
Triglycerides (mmol/l)	1.33 ± 0.63	1.57 ± 0.72	1.38 ± 0.76	0.567	1.52 ± 0.58	1.46 ± 0.79	1.2 ± 0.66	0.536	1.45 ± 0.59	1.44 ± 0.77	1.38 ± 0.8	0.981
Nitrites (µmol/l)	3.94 ± 1.38	4.71 ± 2.54	4.95 ± 1.85	0.402	4.79 ± 1.79	3.67 ± 0.86	4.7 ± 2.57	0.295	4.86 ± 1.87	4.24 ± 2.26	3.95 ± 0.92	0.541

Data are expressed as mean  ±  s.d.

*SCH* schizophrenia, *MetS* metabolic syndrome, *BMI* body mass index, *HDL* high-density lipoprotein, *LDL* low-density lipoprotein

*P < 0.05 vs. CC genotype by one-way ANOVA with Fisher’s LSD post hoc test

**Fig. 1:**
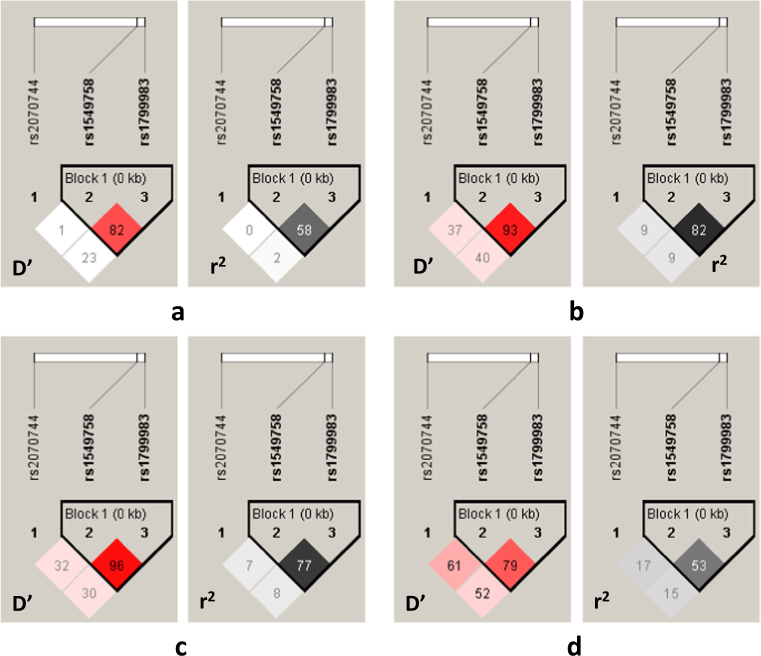
LD plot showing the positions of the three *NOS3* gene polymorphisms in SCH patients with MetS (**a**), normal weight SCH patients (**b**), MetS patients (**c**), and healthy controls (**d**). Values in squares are the pairwise calculation of the LD coefficient *D*′ and the correlation coefficient *r*^2^ expressed in percentage. The color code in *D*′ plots follows the standard color *D*′/logarithm (base 10) of odds (LOD) scheme for Haploview: white, |*D*′| < 1, LOD < 2; shades of pink/red, |*D*′| < 1, LOD greater than or equal to 2. For *r*^2^ plots, different colors designate the extent of LD: for *r*^2^ LD plots white (*r*^2^ = 0), shades of gray (0 < *r*^2^ < 1), black (*r*^2^ = 1)

The LD plot with LD scores (*D*′ and *r*^2^) generated by pairwise comparison of investigated SNPs is shown in Fig. 1. The C774T and G894T SNPs were in strong LD in all samples (*D*′ > 0.7, *r*^2^ > 0.5). Table 5 reflects the frequencies of haplotypes formed by these two NOS3 SNPs. Haplotype analysis showed that the frequency of the 774T/894T haplotype containing both mutant alleles was higher in MetS patients in comparison to healthy controls, and this haplotype was associated with increased occurrence of MetS (*P* = 0.0004, odds ratio = 2.18, 95% CI 1.4–3.37). In turn, the most common 774C/894G haplotype was associated with a decrease in MetS risk (*P* = 0.013, odds ratio = 0.61, 95% CI 0.41–0.9). The distribution of haplotype frequencies between SCH patients with MetS and normal weight SCH patients was not of significant difference.

**Table 5:** Estimated haplotype frequencies and association significance for NOS3 C774T and G894T polymorphisms

Haplotype	Haplotype frequencies		OR (95% CI)	*P* value	Haplotype frequencies		OR (95% CI)	*P* value
	SCH patients with MetS (*n* = 70)	Normal weight SCH patients (*n* = 190)			MetS patients (*n* = 155)	Healthy controls (*n* = 100)		
774C/894G	0.699	0.752	0.77 (0.5–1.18)	0.225	0.628	0.734	0.61 (0.41–0.9)	0.013
774C/894T	0.029	0.011	2.7 (0.68–10.67)	0.756	0.049	0.061	0.81 (0.37–1.75)	0.585
774T/894G	0.058	0.022	2.78 (1.03–7.5)	0.217	0.008	0.031	0.24 (0.05–1.09)	0.229
774T/894T	0.214	0.215	0.99 (0.62–1.59)	0.969	0.315	0.174	2.18 (1.40–3.37)	0.0004

*OR* odds ratio, *CI* confidence interval

## Discussion

In this study three *NOS3* gene polymorphisms were investigated in samples of SCH patients with MetS and normal weight, and of these, only the T-786C SNP was established to be significantly associated with MetS in SCH patients. Our finding contradicts the results of Burghardt et al. [26] that showed that the −786C allele can preserve endothelial functioning only for SCH patients who are not subjected to chronic pro-inflammatory state related to MetS. Similarly, they found no association between endothelial functioning and the G894T polymorphism for SCH patients with or without MetS [26]. The association of the C-786 allele with decreased MetS risk has been shown previously in the Taiwanese general population [20]. Two Japanese investigations have also reported about the association of this allele with a decrease in risk for atherosclerosis and reduced serum triglyceride levels during leisurely physical activities [28, 29]. By contrast, the other Japanese study has revealed that the C allele carriers had both higher blood pressure and lower endothelium-dependent vasodilation when compared to non-carriers; this is consistent with our results [30]. Our results of case−control study in SCH patients are consistent with findings from population-based association studies in different general populations. Associations of TC + CC genotypes of the T-786C SNP with MetS in Koreans and with insulin resistance in Japanese subjects without diabetes have been demonstrated [31, 32]. High-risk haplotypes for MetS susceptibility containing the −786C allele have been established in Arab and Spanish populations [19, 21]. We have explored the associations of NOS3 SNPs with MetS risk in the context of SCH, but similar findings related to the T-786C mutant allele and MetS risk were found in studies in patients with different non-psychiatric disorders, in particular ischemic and non-ischemic cardiomyopathy, hypertension and T2DM [32–34].

According to Nakayama et al. [17] the rare C allele of the T-786C SNP was connected with a reduction in promoter activity of the *NOS3* gene. The molecular mechanism underlying a decrease in promoter activity in individuals carrying the –786C allele might be related to its binding with the replication protein A1. This protein can act as a gene repressor [35]. Two other experiments demonstrated that *NOS3* gene and protein expression levels in human endothelial cells under shear stress conditions were greatly decreased or absent for cells possessing a CC genotype [36, 37]. The association of the TT genotype with lower MetS risk in SCH patients revealed in our study is consistent with other research showing the association of this genotype with the maintenance of endothelium-dependent vasodilation in Caucasian hypertensive patients [38]. In the present study, total cholesterol concentration was lower in SCH patients with MetS who happened to be carriers of TT genotype, in comparison with −786CC homozygotes. The revealed association of the T-786C SNP with total cholesterol levels in SCH homozygous carriers of the harmful recessive alleles may be functionally connected to the development of hypercholesterolemia that accompanies MetS. The association of CC genotype with higher serum total cholesterol has been previously described in Caucasian patients with MetS defined according to IDF criteria [39]. Moreover, modulation of the connection between blood pressure and serum cholesterol within the general population has been reported for G894T variant in *NOS3* gene [40].

The other main outcome from this study is that the haplotype 774C/894T is associated with an increased MetS risk for the Russian population. The C774T polymorphism, located at exon 6, is functionally neutral, because the C to T transition does not result in amino acid substitution. The effect of this haplotype block of high LD formed by C774T and G894T SNPs on MetS susceptibility can likely be attributed to the G894T substitution resulting in a glutamate or aspartate positioned at 298 of eNOS, respectively. The NOS3 G894T polymorphism was correlated with a reduction in basal NO production for healthy subjects [41]. Tesauro et al. [18] have determined that *NOS3* gene product with aspartate, as opposed to glutamate positioned at 298 is likely to cleave in normal tissue and in cells which overexpress eNOS. Other suggested mechanisms for this nonsynonymous mutation are based on the disruption of NOS3 caveolar localization or altered interaction with regulatory proteins including caveolin-1 [42]. Previously, it has been shown that this synonymous SNP in strong LD with the G894T polymorphism was associated with coronary artery disease which is a major adverse consequence of MetS [23, 24]. Considering the disputed data of the effect of the polymorphism in exon 7 on impaired enzyme function, Novoradovsky et al. [43] suggested a hypothesis that both C774T and G894T polymorphisms could be markers of an unknown functional NOS3 SNP, which is in LD with them.

Most studies that report an association of *NOS3* gene variants with MetS aspects have utilized NOS3 SNPs alone to describe genetic architecture. For instance, G894T SNP has been individually associated with the features of MetS in Brazilian, Italian, Tunisian, Taiwanese, and Indian populations [22, 44–48]. Furthermore, haplotype -786C/894G was found to be associated with MetS susceptibility in hypertensive subjects in the Spanish population [34]. Similarly, the G894T SNP in LD with other SNPs was found to be linked to MetS susceptibility in Arab and Taiwanese populations [20, 21]. We revealed no associations for G894T or C774T SNPs with biochemical parameters of lipid and carbohydrate metabolism in MetS patients with or without SCH. However, some other studies demonstrated the associations between G894T SNP and plasma triglycerides in Caucasian patients with obesity and T2DM [49, 50]. Moreover, NOS3 G894T polymorphism is likely a predictor for persistent hyperglycemia for individuals who have a compromised glucose tolerance and atherogenic lipid profile in Asian populations [30, 46, 51].

This study is the first that assesses and compares serum nitrite levels in SCH patients with MetS and normal BMI. Nitrites are thought to be a main area of storage for circulating NO pool. Alterations in plasma nitrites sensitively reflect acute changes in eNOS activity in human forearm circulation [52]. In our work, the observed highly enhanced synthesis of the plasmatic nitrites in SCH patients with MetS compared to normal weight SCH patients might play a compensatory and protective role neutralizing the endothelium-damaging molecular substances. In contrast to the comparison of circulating nitrite levels in SCH samples, no significant changes in serum nitrites were observed between MetS patients and healthy controls. Our findings are consistent with two previous works that concluded that MetS and T2DM appear not to influence nitrite plasma levels [53, 54]. In another study, whole-blood nitrite concentrations in hypertensive obese children and adolescents did not differ from the controls [55].

We found no influence of all three NOS3 SNPs on nitrite concentrations on MetS in SCH or non-SCH subjects. The lack of evidence for the contribution of T-786C and G894T SNPs to circulating nitrite/nitrate levels has been also reported in other studies conducted in healthy subjects [56, 57]. Interestingly, while NOS3 genotypes did not have a significant association with plasma nitrite concentrations in the study of Metzger et al., the NOS3 haplotype including −786C allele and G894 allele did have an association with lower plasma nitrites when compared to those found in other haplotype groups in healthy subjects [58]. However, the same haplotype did not reveal association with nitrite concentrations in hypertensive or normotensive obese children and adolescents [59]. A lack of effects for all three NOS3 SNPs investigated in our study on nitrite concentrations suggests that these SNPs possibly promote MetS by other mechanisms, thus far undetermined.

Certain limitations of this study should be considered. A major limitation might be the fact that described case−control studies have been conducted in two different Russian regions (Siberia and Kaliningrad Region). However, subjects in all samples were self-reported Caucasian Russian, and all cases were ethnically and geographically matched with control subjects within each case−control comparison. Another limitation of our work includes the somewhat small sample sizes. We also could not exclude the possibility of other factors affecting our results, such as individualized effects of smoking, or the fact that our study contained a higher proportion of women than men in the SCH with MetS group compared to the normal weight SCH group. Finally, the subjects in this study did not have a specific diet prior to blood sampling. Although possible increased dietary nitrate may lead to increased circulating nitrite concentrations, it has been shown that in adults, most plasma nitrites are derived from the oxidation of eNOS-derived NO [60]. Thus, due to the limitations described above, our results should be considered preliminary. To confirm the findings from our study, future studies that have larger samples are necessary. In conclusion, the findings from the study indicate that the NOS3774T/894T haplotype is significantly associated with greater MetS risk than each of the individual harboring alleles considered alone. However, in the presence of SCH and such factors as antipsychotic therapy, G894T and C774T variants probably do not contribute to MetS risk, while the NOS3 T-786C polymorphism seems to be a determining factor. The association of this promoter polymorphism affecting the transcription rate of the *NOS3* gene with serum total cholesterol might be a possible mechanism by which the T-786C SNP affects the development of MetS features in SCH subjects.

### Data availability

Data are available in the NCBI ClinVar database (http://www.ncbi.nlm.nih.gov/clinvar/) under accession numbers SUB3605688: SCV000680062 and SUB3729182: SCV000692592.
